# A Reduced Number of mtSNPs Saturates Mitochondrial DNA Haplotype Diversity of Worldwide Population Groups

**DOI:** 10.1371/journal.pone.0010218

**Published:** 2010-05-03

**Authors:** Antonio Salas, Jorge Amigo

**Affiliations:** 1 Unidade de Xenética, Departamento de Anatomía Patolóxica e Ciencias Forenses, and Instituto de Medicina Legal, Facultade de Medicina, Universidade de Santiago de Compostela, Galicia, Spain; 2 Grupo de Medicina Xenómica, Universidade de Santiago de Compostela, Galicia, Spain; University of Glasgow, United Kingdom

## Abstract

**Background:**

The high levels of variation characterising the mitochondrial DNA (mtDNA) molecule are due ultimately to its high average mutation rate; moreover, mtDNA variation is deeply structured in different populations and ethnic groups. There is growing interest in selecting a reduced number of mtDNA single nucleotide polymorphisms (mtSNPs) that account for the maximum level of discrimination power in a given population. Applications of the selected mtSNP panel range from anthropologic and medical studies to forensic genetic casework.

**Methodology/Principal Findings:**

This study proposes a new simulation-based method that explores the ability of different mtSNP panels to yield the maximum levels of discrimination power. The method explores subsets of mtSNPs of different sizes randomly chosen from a preselected panel of mtSNPs based on frequency. More than 2,000 complete genomes representing three main continental human population groups (Africa, Europe, and Asia) and two admixed populations (“African-Americans” and “Hispanics”) were collected from GenBank and the literature, and were used as training sets. Haplotype diversity was measured for each combination of mtSNP and compared with existing mtSNP panels available in the literature. The data indicates that only a reduced number of mtSNPs ranging from six to 22 are needed to account for 95% of the maximum haplotype diversity of a given population sample. However, only a small proportion of the best mtSNPs are shared between populations, indicating that there is not a perfect set of “universal” mtSNPs suitable for all population contexts. The discrimination power provided by these mtSNPs is much higher than the power of the mtSNP panels proposed in the literature to date. Some mtSNP combinations also yield high diversity values in admixed populations.

**Conclusions/Significance:**

The proposed computational approach for exploring combinations of mtSNPs that optimise the discrimination power of a given set of mtSNPs is more efficient than previous empirical approaches. In contrast to precedent findings, the results seem to indicate that only few mtSNPs are needed to reach high levels of discrimination power in a population, independently of its ancestral background.

## Introduction

Variations in human mtDNA molecules have been deeply investigated in several fields of biomedical research such as forensic genetics, molecular anthropology, and disease studies [1], [2], [3], [4], [5], [6], [7], [8], [9], [10], [11], [12], [13], [14], [15], [16], [17], [18], [19]. There are 100 to 10,000 copies of the mtDNA genome per cell, and each of them consists of circular molecules of about 16,569 base pairs (bps). The mtDNA is inherited exclusively from the mother. Each mtDNA genome can be divided into two main parts: the control and the coding region. The control region occupies about 1,200 bp of the molecule and contains, among other, regulatory elements related to the replication of mtDNA or gene expression. It is usually divided into two segments characterised by their high (average) mutation rate, namely the first and second hypervariable segments (HVS-I/II). The coding region encodes 37 densely packed genes (13 for proteins, 22 for transfer RNA [tRNA], and two for subunits of ribosomal RNA [rRNA]) that are needed to maintain the correct function of the mitochondrion.

The study of mtDNA variability has been approached using different methodologies. Analysis of restriction fragment length polymorphism (RFLP) sites and screening procedures such as heteroduplex analysis (HD) and single strand conformation polymorphisms (SSCP) have been extensively used in the past and are still used by many laboratories [2], [20], [21]. Since the mid-1990s, sequencing the HVS-I (and sporadically the HVS-II), coupled with the analysis of selected RFLP sites, has been the most common strategy for analysing mtDNA variation. Nowadays, there are more than 130,000 HVS-I mtDNAs from different human population groups reported in the literature.

The interest in analysing complete mtDNA genomes is growing as indicated by the more than 6,700 complete genomes available in the literature and in GenBank, most of them reported in the past five years. Sequencing complete genomes is now benefiting from improvements to the sequencing chemistry and the higher sophistication of automatic sequencers. However, this analysis is technically complex and costly and, therefore, unfeasible not only for most biomedical applications at high-throughput scales, but also for those applications depending on low quality or small amounts of DNA (e.g., forensic samples). This is the main reason why the majority of laboratories just target the control region (usually the HVS-I), complemented by analysing selected coding region SNPs. The traditional screening approaches (RFLP, SSCP) for genotyping mtSNPs are now being replaced by lower cost mini-sequencing techniques that allow multiplexing several polymorphisms in single reactions [22], [23], [24], [25]. In particular, forensic geneticists are especially interested in developing mini-sequencing assays that interrogate a reduce numbers of mtSNPs per reaction (e.g., multiplexes of five to 20 mtSNPs), because the technique can perform well with degraded or low copy number samples [26]; many evidentiary samples only allow a single PCR reaction, and high-throughput mtSNP techniques are unsuitable for sub-optimal samples [27]. Ideally, targeted mtSNPs should retain the maximum level of discrimination power in a single mtDNA test and, therefore, a careful selection of mtSNPs for mini-sequencing assays is a key step in the process. Generally, this selection is based on phylogenetic criteria, where mtSNPs are chosen from the phylogeny to represent the main branches of the phylogenetic tree (which define haplogroups); however, this strategy does not necessarily optimise the discrimination power of a particular set of mtSNPs. Alternatively, mtSNPs can be selected according to their mutation rate; mtSNPs with the highest mutation rates tend to yield the maximum diversity values. However, current positional mutation rates are under suspicion because the methods employed to compute them could be flawed [28], [29], and only recently a new proposal of site-specific mutation rates has been published aimed at overcoming the problems of past approaches [29]. It is impossible to decide *a priori* which one of these two approaches is more efficient for maximizing discrimination power. It is possible that a combination of both rationales could perform better. On the other hand, a particular combination of mtSNPs could yield good results in a specific population context, but might be unsuitable in a different population group (say Europeans *versus* Native Americans). Ideally, we could also envisage selecting a universal mtSNP panel that could generate reasonable discrimination power independently of the population group considered. Moreover, this panel would be particularly useful when dealing with highly admixed populations (e.g., the US, South American admixed populations, highly cosmopolitan cities).

With the latter applications in mind, we aimed to explore the optimum combinations of mtSNPs needed to maximize the diversity values of a given population group, taking into account the premise that multiplexing techniques (excluding high-throughput platforms) only allow genotyping a moderate number of mtSNPs (usually a maximum of 20 to 30). The method employed here is based on an algorithm that allows the exploration of the full set of combinations arising from a given set of known mtSNPs. We then evaluate the combinations yielding the highest values of diversity and the best candidate mtSNPs within these combinations. Various biomedical applications will also be discussed.

## Material and Methods

### Complete genome database

The database used for the simulation experiments was built based on the following criteria:

- Selecting complete genomes from the literature and GenBank capable of being used as proxies of human population samples representing main continental regions. This criterion filters out those available complete genomes that have been analysed based on phylogenetic criteria or, in particular, patients in (mtDNA) disease studies [1], [30], [31], [32], [33], [34], [35], [36];
- The main population groups should be represented by at least 300 complete genomes such that most of the considered ‘speedy’ mutations (*sensu* [37]) can be polymorphic with a minimum allele frequency (MAF) >5% in the whole database; and
- The compiled database should represent at least three main continental groups, namely, Africans, Asians, and Europeans.

According to these criteria, we collected the following datasets: (i) *N* = 309 from [5], representing the African subset; (ii) *N* = 672 Japanese to represent the Asian subset [38]; and (iii) *N* = 241 from [24] and *N* = 192 [39] representing the European subset. In addition, *N* = 326 individuals belonging to the American haplogroups A2, B2, C1, D1, and X2a (see [3], [10] and references therein) were also collected for representing a Native American subset. Apart from the mentioned relatively homogenous population groups, two admixed population samples from the US, ‘African-American’ and ‘Hispanic’ datasets (*N* = 140 and *N* = 125 respectively) from [40], were also used in the simulation experiments.

We are aware of potential sequencing errors affecting some of these datasets and have tried to disregard suspicious complete genomes or use corrected versions of the reported datasets (not necessarily those available in GenBank) [29], [41]. For instance, some of the Tanaka's complete genomes were corrected in Kong et al. [42] but the flawed versions of these genomes are still in GenBank [41].

As usual, mtSNPs are referred using the revised Cambridge Reference Sequence or rCRS [43].

### Panel of SNPs

The approach followed in this study is based on exploring all (or a subset of all) possible combinations that arise from combining a given set of *n* mtSNPs candidates taken *m* at a time (in what follows, *m* value). Given the fact that the number of mtSNP variants considered in this study is above 3,200, it is computationally impossible to explore the entire universe of combinations arising from such a large number of variants (for instance, the number of possible combinations *C*(100,20) is >10^22^). To overcome this problem, we selected the 394 mtSNPs from the whole dataset fulfilling a MAF >0.05. By definition this criterion eliminates rare mtSNPs (MAF≤0.05) under the premise that these SNPs cannot substantially contribute to increase diversity levels of populations. These variants are mainly ‘private’ to single genomes (singleton mutations usually located at the tips of the mtDNA phylogenies); therefore, these variants cannot be extrapolated to independent or larger samples due to ascertainment bias. On the contrary (see above), the selected mtSNPs (and in particular those that better contribute to increase the discrimination power) are polymorphic in different human populations; either (i) because they mutated before the divergence of major population groups, or (ii) because they have a high mutation rate (mutational hotspots). Variants that are known to be problematic from a genotyping point of view were disregarded (16182C, 16183C, 16193+C, variants around 310, length variation around positions 523–524, etc.).

### Programming

For the panel of mtSNPs considered and all possible *m* values, we computed two diversity indices in every population dataset: (a) the ‘normalised’ number of haplotypes, defined as *H* =  *h*/*N*, where *h* represents the number of different haplotypes in the dataset defined by a given *m* and *N* is the dataset sample size; and (b) the haplotype diversity, defined as *HD* = 1 − 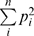, where *p*_i_ is the haplotype frequency of the *i* haplotype resulting from each *m* combination of mtSNPs. We are aware to the fact that *H* does not scale out the dependence on sample size [44], [45], [46], and therefore the results on *H* cannot be extrapolated across samples of different sizes.

The main drawback of this study is the extremely high computational cost needed to explore the entire universe of all possible mtSNP combinations. We run a parallelisable script on a shared memory node system with the SMP NUMA architecture and a cluster containing over 2500 processors named *Finis Terrae*, which is available at the Supercomputing Centre of Galicia (CESGA; http://www.cesga.es/). Considering that studying each of the *m* values is completely independent from the rest, we dispatched each individual analysis run among the available cores of the cluster, reducing the overall running time to the largest *m* value analysis as if performed alone.

We ran up to 10,000 iterations for each *m*. We recorded the values of *H* and *HD* and derived their mean and standard deviation values. We also recorded the maximum values of diversity obtained for each *m* among the full number of iterations.

For every dataset, we also computed the maximum diversity values (*H_MAX_* and *HD_MAX_*) that could be obtained considering the complete genome information.

## Results

### Considerations about computational limitations

The large amount of mtSNPs considered in the different mtSNP panels tested made it computationally unfeasible to explore all possible combinations of *n* mtSNPs taken *m* at a time. We, therefore, developed an algorithm that creates subsets of *m* mtSNPs by sampling without replacement and equal probability each studied panel, while recording the best mtSNP combinations for every *m* value (the ones giving the maximum diversity values of *H* and *HD*). Other sampling strategies were used, such as those based on a stepwise mtSNP selection (find the best single mtSNP and then the best one to add it, and so on) but the one presented here was the most efficient in terms of maximizing the values of *H* and *HD* (data not shown). However, we first assessed the impact of analysing only a given number of combinations (iterations) of mtSNPs on the estimation of the parameters of interest. We observed that the trend in *H* or *HD* values with different *m* values was reasonably stable when exploring at least 10,000 mtSNP combinations (Figures S1, S2, S3, S4, S5). Based on this simulation, we decided to run 10,000 iterations for every population scenario considered in this study.

**Figure 1:**
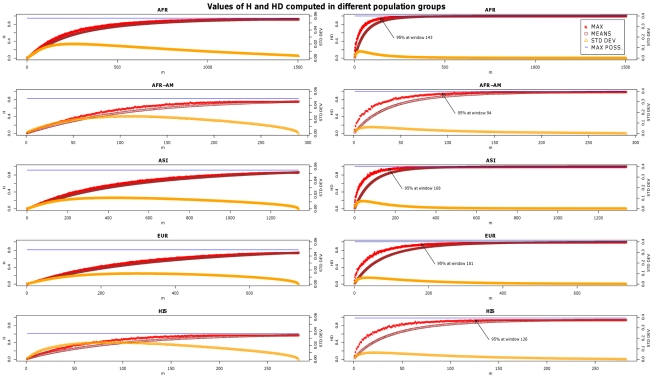
Values of *H* (left panel) and *HD* (right panel) computed in different population groups. The x-axis represents the numberofmtSNP considered. The top right legend indicates the colour codes for the maximum values of *H* and *HD* (max), the means, standard deviations, and maximum possible value of *H* and *HD* (computed assuming the full information provided by the complete genomes in each population dataset). Codes for populations are AFR  =  Africans, AFR-AM  =  ‘African-Americans’, ASI  =  Asians, EUR  =  Europeans, and HIS  =  ‘Hispanics’.

We are aware that this approach will probably prevent capture of the best candidate combination of mtSNPs (the one providing the highest real value of *H* and *HD*), but this is not the main goal of the simulations. The simulation approach could be considered a success if the best combination of mtSNPs obtained from it performed much better than traditional procedures. On the other hand, note that the standard deviation values of *H* and *HD* for the different *m* values (Figure 1) were very low, indirectly indicating not only that these values do not fluctuate significantly on different iterations, but also that the best values should be close to the ones obtained using 10,000 iterations.

### Performance of different mtSNP combinations on diversity values

The shapes of the *H* and *HD* curves in Figure 1 respond to the same expected phenomenon: increased values of *m* are accompanied by progressive increments in the values of *H* and *HD*. However, the *m* values needed to reach a *plateau* are different for *H* and *HD*. While *H* grows slowly by increasing the number of mtSNPs analysed, *HD* reaches its maximum quickly at lower mtSNP *m* values. Thus, somehow unexpectedly, the *HD* curves (Figures 1) indicate that only a small number of mtSNPs is required to saturate the diversity values to 95% of the *HD_MAX_* independently of the population database employed (Figures 1 and Table 1). In other words, the addition of new mtSNPs beyond a certain point does not apparently contribute to an increase in discrimination power.

**Table 1:** Diversity values (*H* and *HD*) for the SNPs considered in different articles published in the literature and comparison with those obtained in the present study.

		AFR			AFR-AM			ASI			EUR			HIS			ALL		
Study	n° SNPs	N	H	HD	N	H	HD	N	H	HD	N	H	HD	N	H	HD	N	H	HD
Brandstätter et al. (2003)	16	9	0.0485	0.5660	6	0.0571	0.3830	6	0.0223	0.7153	15	0.0647	0.9155	10	0.0640	0.5533	15	0.0270	0.7991
Vallone et al. (2004)	11	4	0.0189	0.5217	2	0.0214	0.4033	5	0.0104	0.7508	11	0.0416	0.8242	3	0.0400	0.6485	11	0.0138	0.7343
Quintáns et al. (2004)	17	10	0.0404	0.1789	3	0.0286	0.1099	7	0.0134	0.7446	16	0.0416	0.9075	8	0.0640	0.7830	16	0.0224	0.8661
Umetsu et al. (2005)	36	15	0.0674	0.7471	8	0.0714	0.6737	31	0.0670	0.9453	21	0.0600	0.8975	14	0.1040	0.8252	36	0.0563	0.9583
Grignani et al. (2005)	16	7	0.0216	0.2530	1	0.0143	0.0826	5	0.0104	0.5214	13	0.0370	0.6030	2	0.0240	0.3435	13	0.0103	0.4723
Brandstätter et al. (2006)	45	21	0.0755	0.7191	6	0.0500	0.5253	13	0.0298	0.7313	34	0.0878	0.8803	6	0.0560	0.5415	40	0.0419	0.7843
Wiesbauer et al. (2006)	10	6	0.0296	0.3764	3	0.0286	0.0564	4	0.0119	0.4848	10	0.0370	0.8424	7	0.0640	0.6805	10	0.0155	0.6868
Lee et al. (2006)	22	9	0.0350	0.2713	4	0.0357	0.1497	21	0.0446	0.9225	7	0.0208	0.5636	9	0.0800	0.8210	22	0.0281	0.8875
Álvarez-Iglesias et al. (2006)	32	15	0.0755	0.6019	5	0.0429	0.1876	29	0.0670	0.8630	13	0.0393	0.5692	19	0.0960	0.8068	32	0.0534	0.9052
Coble et al. (2004)	59	31	0.2399	0.9410	14	0.1429	0.8558	30	0.0848	0.8889	57	0.1894	0.9463	12	0.1520	0.8810	57	0.1275	0.9504
Endicott et al. (2006)	20	7	0.0216	0.3326	2	0.0214	0.1220	5	0.0089	0.0666	1	0.0046	0.0046	1	0.0160	0.0160	11	0.0069	0.1160
Köhnmenn et al. (2008)	22	13	0.0836	0.7832	7	0.0714	0.6091	9	0.0387	0.8504	22	0.0993	0.9428	11	0.0800	0.7631	22	0.0477	0.8971
Wu et al. (2008)	10	9	0.0512	0.6350	3	0.0286	0.3952	10	0.0253	0.8171	9	0.0208	0.5736	9	0.0640	0.7621	10	0.0213	0.8771
Watkins et al. (2008)	32	16	0.0701	0.5872	5	0.0429	0.4317	19	0.0298	0.7736	21	0.0439	0.7730	15	0.0960	0.7866	28	0.0373	0.9036
Rosa et al. (2008)	19	11	0.0755	0.6174	6	0.0571	0.3725	12	0.0461	0.8394	18	0.0600	0.8978	12	0.0880	0.7930	19	0.0442	0.8811
Present study	9/22/11/10/10	184	0.8679	0.9990	189	0.5714	0.9885	100	0.5774	0.9950	88	0.4111	0.9909	97	0.4960	0.9772	195	0.5325	0.9977
Maximum possible values	–	–	0.9569	0.9997	–	0.9286	0.9990	–	0.9435	0.9998	–	0.8545	0.9989	–	0.7280	0.9876	–	0.9075	0.9998

Thus, the mtSNPs reported in the different published panels are used to compute *HD* and *H* in the complete genomes considered in the present study. In the row labelled as “Present study” we indicate the number of mtSNPs needed to cover at least 95% of the maximum *HD* in the different population groups; numbers in the second column separated by slash correspond to AFR (Africans), AFR-AM (‘African-Americans’), ASI (Asians), EUR (Europeans), and HIS (‘Hispanics’), respectively. The final column headed as ALL refer to the values after lumping the full sub-sets of complete genomes. The bottom row indicates the maximum possible values of *H* and *HD* for the different population groups considering the whole genome information.

The mathematical difference between *H_MAX_* and *HD_MAX_* and the mean values of *H* and *HD* are more pronounced for intermediate *m* values (Figures 1) because these *m* values admit more different combinations of mtSNPs, thereby yielding a wider range of *H* and *HD* values. Concordant with this observation is the shape of the standard deviations, indicating that the maximum fluctuations of *H* and *HD* values also occur around these intermediate *m* values.

### Evaluation of the best candidate mtSNPs

All the mtSNPs were ranked in a list according to the number of times each of them appears in a combination that maximizes the *HD* values; the first position being assigned to the one that appears most often (Table S1).

To some extent, the best mtSNPs in the different population groups considered in this study overlap (Table S1 and Table S2). For instance, transition T16519C appears to be the best mtSNP in virtually all the different population groups. Other well-known mutational hotspots, T152C, T195C, T16189C, and T16311C, occupy different positions in the ranking (which in part mirrors the stochastic nature of the simulation approach used in the present study), but all are listed among the top 15 top mtSNPs in the three main continental groups when looking at the *HD* values (Table 2 and S1).

**Table 2:** Excerpt of the data in Table S1 showing the top five mtSNP that are shared between the top 15 mtSNPs in the three main continental groups.

Position	AFR	AFR-AM	ASI	EUR	HIS	ALL	rCRS	Variant	MapLocus	MR
16519	1	1	1	1	1	1	T	C	MT-DLOOP1	1
152	2	3	4	2	5	2	T	C	MT-DLOOP2	2
16189	3	7	2	6	14	3	T	C	MT-DLOOP1	6
16129	6	115	3	8	6	4	G	A	MT-DLOOP1	7
195	7	44	9	14	0	8	T	C	MT-DLOOP2	5

MR (mutational ranking) column refers to the position of these variants in the list of relative site-specific mutation rates as reported in [29]. Other legends are as in Table S1.

The scores in Table S1 are useful for those analysts (e.g. forensic geneticists) wishing to empirically design a test panel of mtSNPs in a given population context. Thus, for instance, from Figures 1 (and Table 1), we know that nine mtSNPs sufficiently account for 95% of the haplotype diversity in a given African sample. From Table S1, it can be observed that the top nine polymorphisms in the African sample are for the values on *HD* (sorted by their position in the ranking): T16519C, T152C, T16189C, A189G, T16093C, G16129A, T195C, T16311C, and G143A.

### Diversity accounted by existing mtSNP panels and the simulation-based approach

A number of different mtSNP panels have been proposed in the literature to date, and these provide a suitable framework for evaluating the efficiency of the simulation-based approach used in this study.

Table 1 summarises the values of *H* and *HD* for the different mtSNP panels proposed in the literature and those used in this study, all of them evaluated using the same collections of complete genomes (see M&M). Some of the panels reported in the literature were designed for specific population groups, mainly Europeans and Asians, so these panels behave worse in population samples with different genetic backgrounds. For instance, the panel of 45 mtSNPs proposed by Brandstätter et al. [25] was designed to increase the discrimination power within the typically European haplogroup H, but the discrimination power of these mtSNPs in Asians or Native Americans is much lower (Table 1). The opposite example is the mtSNP panel provided in Álvarez-Iglesias et al. [47].

The results summarised in Table 1 clearly indicate that almost all the panels proposed in the literature yield lower values of *H* and *HD* than the mtSNPs combinations inferred from the present study (with the caveat that *H* is not appropriate for inter-population comparisons). In addition, the number of mtSNPs needed to reach 95% of the *H_MAX_* and *HD_MAX_* are very low for most of the population groups (ranging from 10 to 22); while all the panels available in the literature yield values of diversity well below 95% (Table 1).

### Evolutionary nature of the most discriminating mtSNPs

Not surprisingly, the top five mtSNPs that better contribute to increase the diversity values in the different population groups are non-synonymous or are located in non-coding or un-translated regions of the mtDNA molecule (Table 2). In other words, these mtSNPs are almost polymorphic in different human population groups (universal) because they do not seem to be as subjected to the effect of stabilizing or purifying natural selection as coding region variants. These top mtSNPs are in fact located in the control region (non-coding), are transitions, and roughly match the top mutational hotspots reported by [29] (Table 2).

## Discussion

Several fields of research (population, medical, and forensic genetic) are interested in the analysis of mtSNPs for several genetic applications. Some of these applications rest on the ability of a selected (low) number of mtSNPs providing a high discrimination power when analysing human population or casework evidentiary samples. We have used a simulation-based approach to evaluate the discrimination power of mtSNPs in different population contexts, including samples representing the main continental regions and admixed population groups.

Our simulated approach indicates that no more than a dozen mtSNPs is sufficient to account for ∼95% of the maximum level of *HD* diversity for almost all population groups. However, admixed populations, such as ‘African-Americans’, need to double this amount (∼22 mtSNPs) to reach similar values of diversity. The top mtSNP variants are mutational hotspots mainly located in the control region. In addition, only a small proportion of the best mtSNPs are shared between population groups, indicating thatthere is not a perfect set of ‘universal’ mtSNPs suitable for all population contexts.

Today, there are two well-known strategies commonly used for selecting mtSNPs aimed to account for the highest levels of diversity in population groups, namely, the phylogenetic-based approach and mtSNP selection based on mutational hotspots. The results of the present study seem to indicate however that these traditional strategies perform worse than the simulation-based approach developed in the present study, and that in reality, it is a combination of highly mutable mtSNPs and haplogroup diagnostic sites that optimized the ability of a given mtSNP panels to account for the highest levels of diversity.

The strategy proposed in this study is particularly relevant for forensic studies, where small panels of mtSNPs are frequently demanded in routine casework, but we can also foresee other biomedical applications. For instance, mtSNP panels could be used to evaluate mtDNA instability in studies on tumours [48], [49], [50], where patients belong to different population groups.

Some caveats should be added about the potential bias arising from the limited number of complete genomes considered in this study. Some potential ascertainment bias could arise when computing *HD* using a limited number of complete genomes. The reproducibility of these results will only be possible with the availability of independent complete genome datasets. A bootstrap strategy could be used instead, but the high computational demands of this procedure makes it unfeasible. However, it can tentatively be said that there are few considerations that allow us to predict reproducible results when applied to independent complete genome datasets. These considerations are that (a) the best mtSNPs have by definition an MAF above 5%, (b) various of the best mtSNPs overlap in different population groups, and (c) most of the best mtSNPs have a high mutation rate. Therefore, these mtSNPs appear in different parts of the worldwide phylogeny and are not restricted to any particular population or ethnic group.

This study has demonstrated that future proposals for mtSNP panels aimed at obtaining a high discrimination power could be considered in the light of the simulation approach proposed here. The phylogenetic approach, although essential for most mtDNA studies (e.g. [19], [51], [52], [53]), is probably not the best tool for predicting the discrimination power of a particular set of a mtSNP panel, but can still be useful for understanding the biological nature of a selected panel of mtSNPs and assist in its design. The mtSNP panels proposed in the literature do not perform as well as those suggested by this study.

## Supporting Information

Figure S1 Effect of size iteration (number of mtDNA combinations explored from the full universe of possible combinations) for the estimation of H and HD in the African dataset. Only the mtSNPs overlapping in all the population datasets were used.
(0.31 MB TIF)

Figure S2 Effect of size iteration for the estimation of H and HD in the ‘African-American’ dataset. Only the mtSNPs overlapping in all the population datasets were used.
(0.25 MB TIF)

Figure S3 Effect of size iteration for the estimation of H and HD in the Asian dataset. Only the mtSNPs overlapping in all the population datasets were used.
(0.29 MB TIF)

Figure S4 Effect of size iteration for the estimation of H and HD in the European dataset. Only the mtSNPs overlapping in all the population datasets were used.
(0.27 MB TIF)

Figure S5 Effect of size iteration for the estimation of H and HD in the ‘Hispanic’ dataset. Only the mtSNPs overlapping in all the population datasets were used.
(0.24 MB TIF)

Table S1 Scores of all the mtSNPs in the different population samples (AFR  =  Africans, AFR-AM  =  “African-Americans”, ASI  =  Asians, EUR  =  Europeans, and HIS  =  “Hispanics”; ALL  =  all the complete genomes considered as a single group) based on HD. First, the number of times a particular mtSNP shows up when computing the maximum values of HD in every iteration and m values is recorded. The mtSNPs are sorted from those that appear more times in the different iterations to those that never appear. The final score is given according to their relative position in this ranking; from 1 (received by the best mtSNP) to n (number of mtSNP in each panel). The columns indicate, in this order, the position of the mtSNP according to the rCRS [48], the mutational change (all are transitions unless a suffix indicates a transversion or an indel specified), the population sample set (as indicated above), the rCRS variant, the nature of the mutational change (transition, transvertion, or indel), the MapLocus, a shorthand of the locus, the locus description, the coding or non-coding condition of the mutational change, the changed-codon, the amino acid change, the relative position of the mtSNP within genes, the relative position of the mtSNP within codons, and their synonymous or non-synonymous condition.
(0.12 MB XLS)

Table S2 Scores of all the mtSNPs in the different population samples based on H. More details in legend of Table S1.
(0.12 MB XLS)
