# Supplementary figures and images for: A Reduced Number of mtSNPs Saturates Mitochondrial DNA Haplotype Diversity of Worldwide Population Groups
Source: PLoS One. 2010 May 3;5(5):e10218. doi: 10.1371/journal.pone.0010218 (PMC2862705; doi:10.1371/journal.pone.0010218)

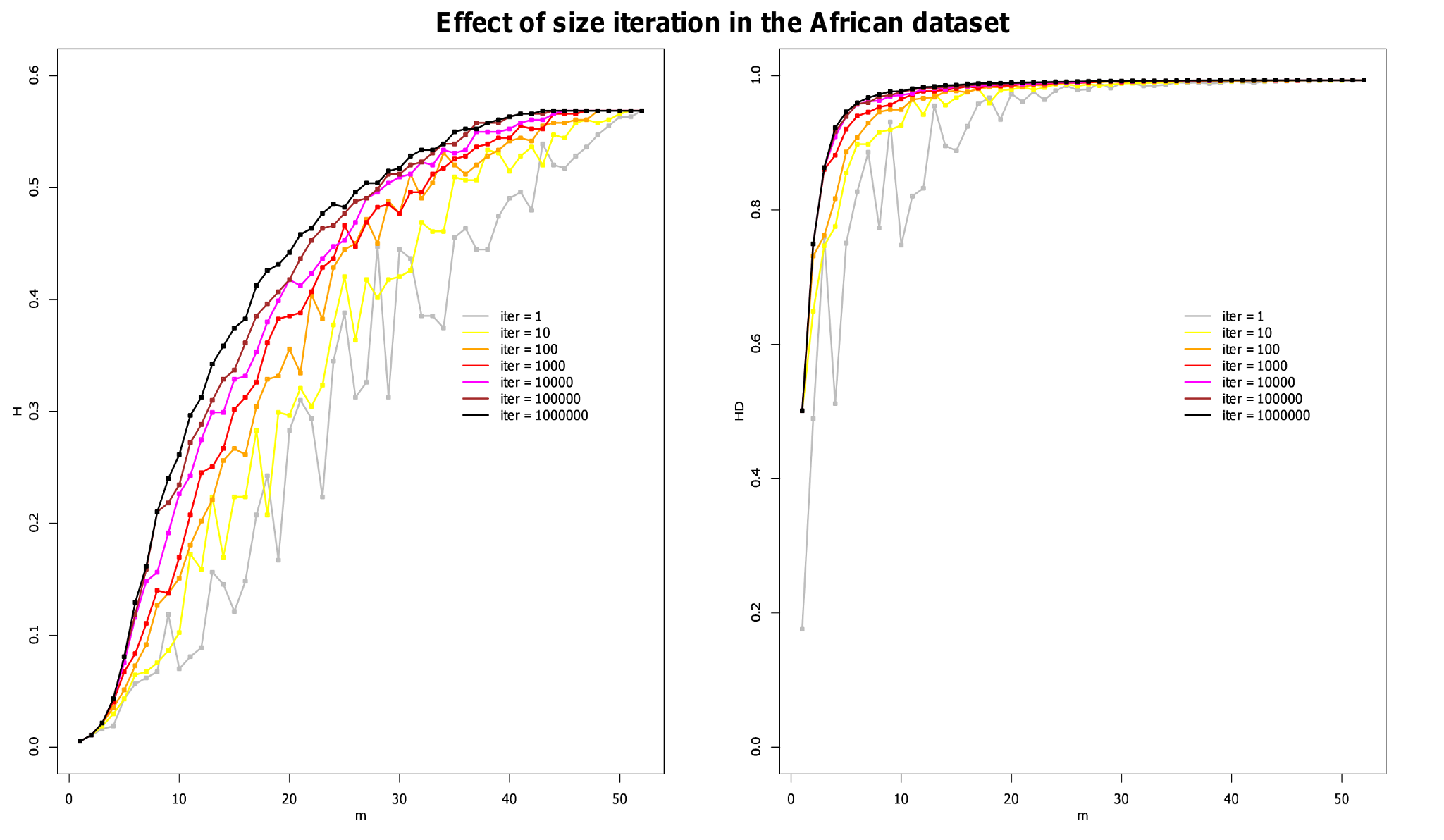

Supplement: Figure S1 — Effect of size iteration (number of mtDNA combinations explored from the full universe of possible combinations) for the estimation of H and HD in the African dataset. Only the mtSNPs overlapping in all the population datasets were used. (0.31 MB TIF) [file pone.0010218.s001.tif]

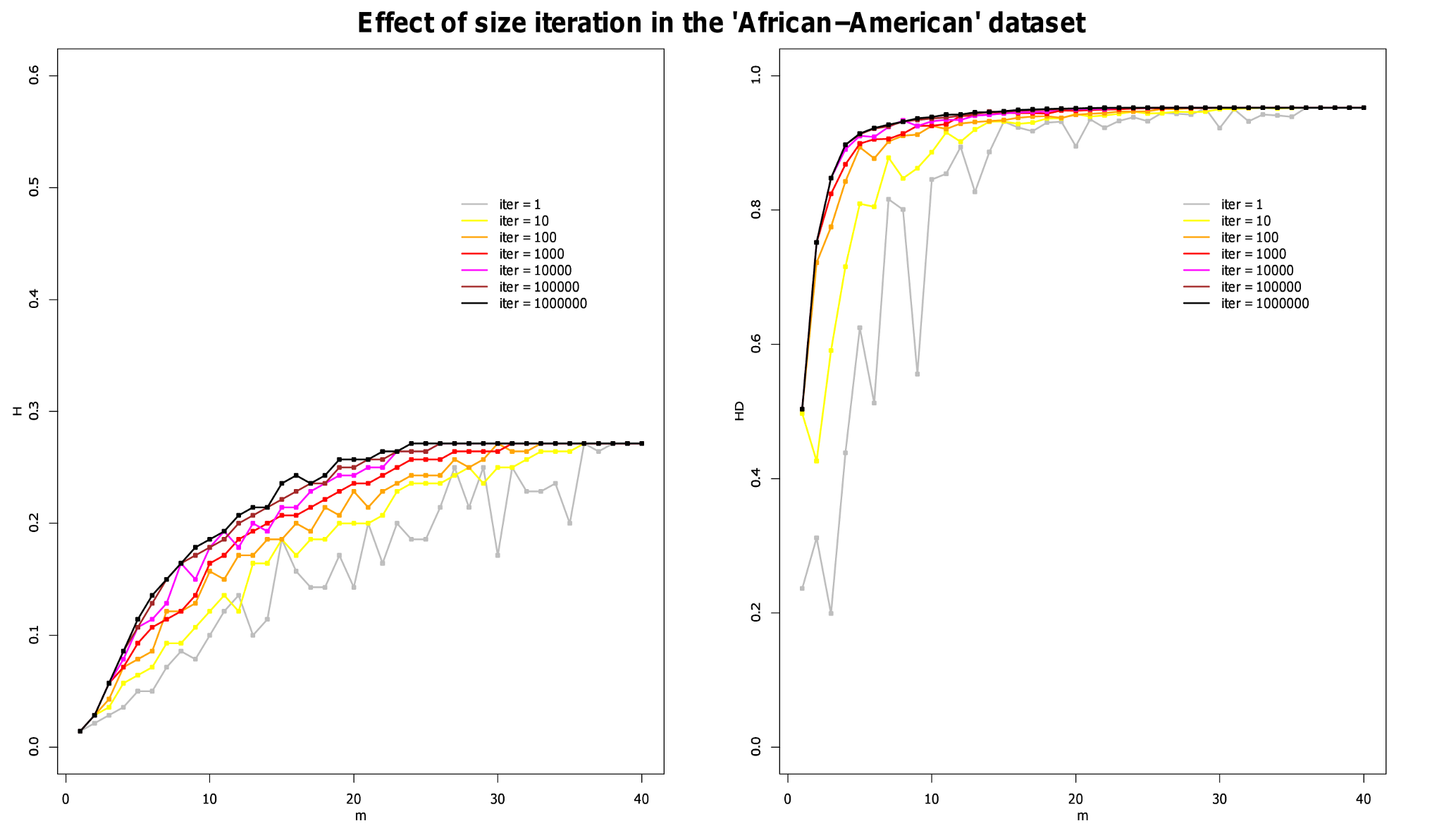

Supplement: Figure S2 — Effect of size iteration for the estimation of H and HD in the ‘African-American’ dataset. Only the mtSNPs overlapping in all the population datasets were used. (0.25 MB TIF) [file pone.0010218.s002.tif]

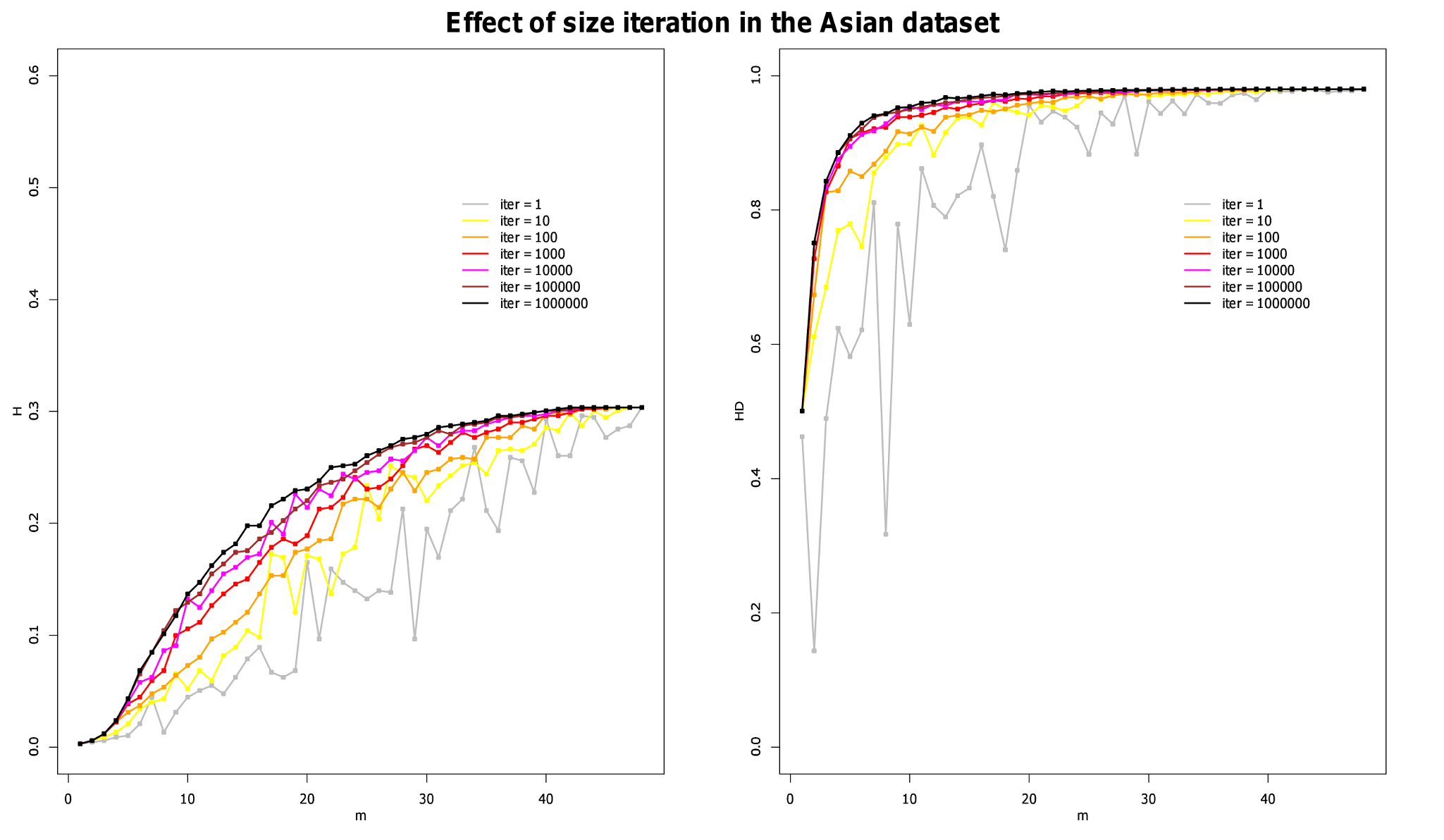

Supplement: Figure S3 — Effect of size iteration for the estimation of H and HD in the Asian dataset. Only the mtSNPs overlapping in all the population datasets were used. (0.29 MB TIF) [file pone.0010218.s003.tif]

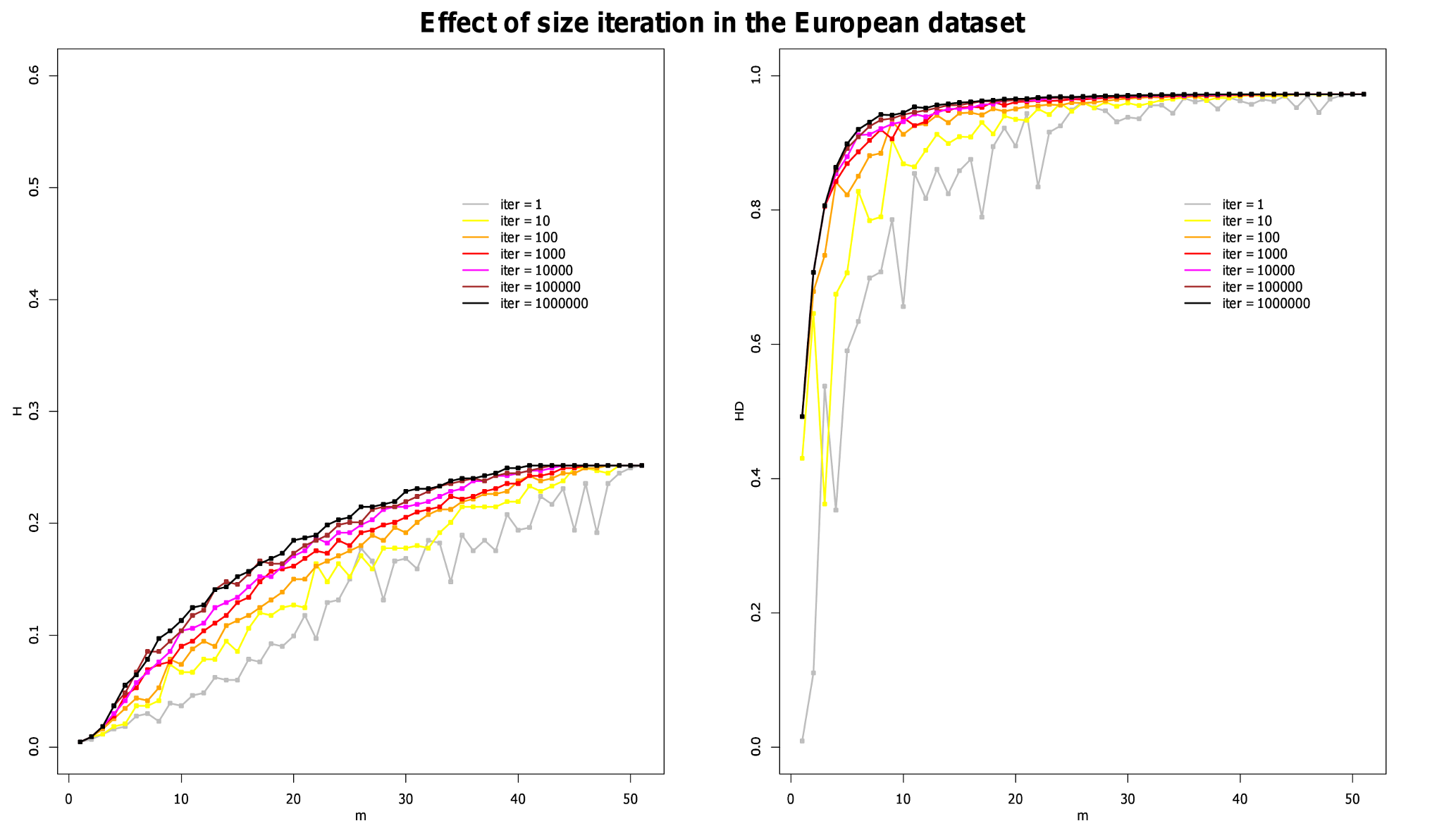

Supplement: Figure S4 — Effect of size iteration for the estimation of H and HD in the European dataset. Only the mtSNPs overlapping in all the population datasets were used. (0.27 MB TIF) [file pone.0010218.s004.tif]

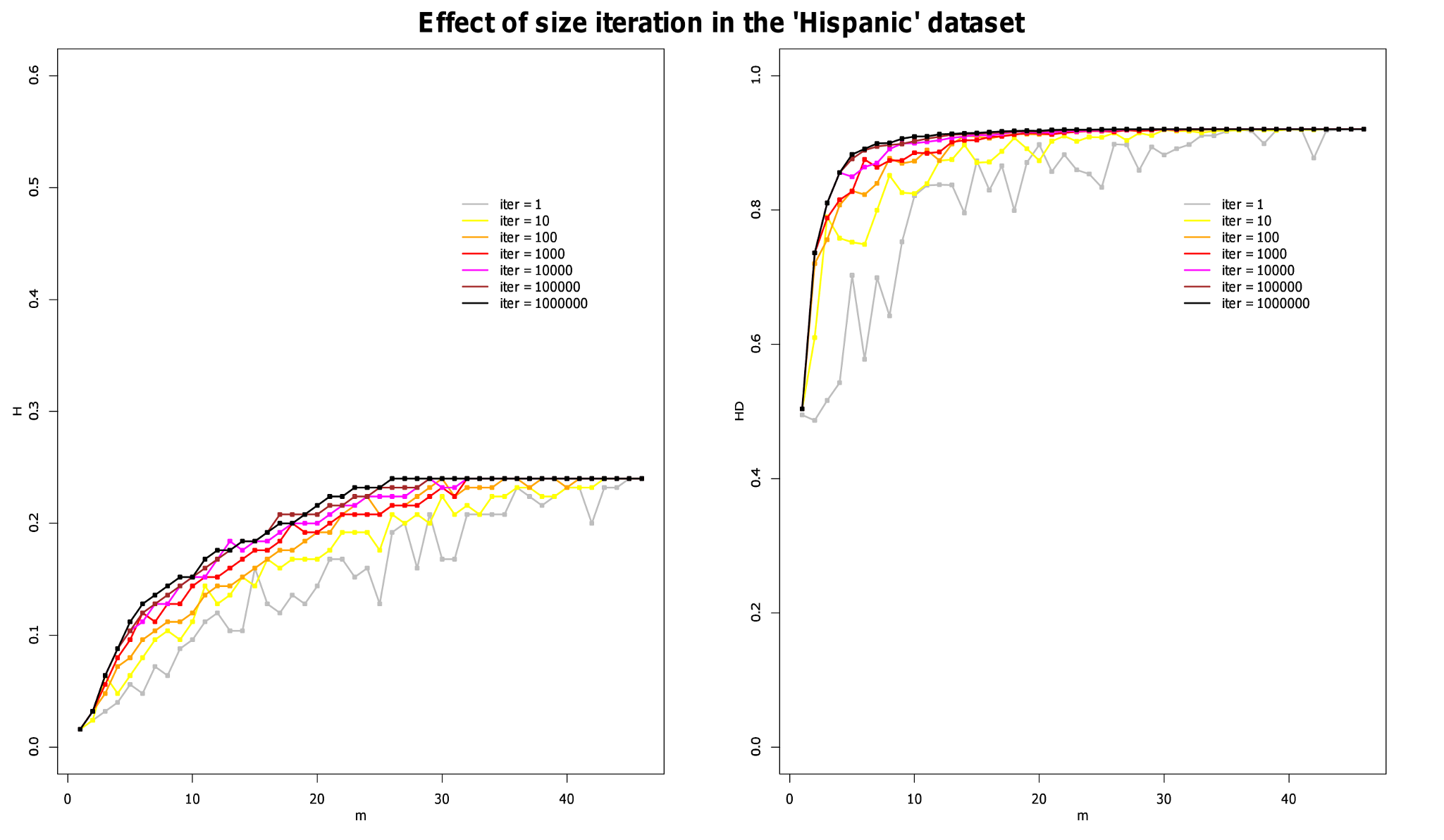

Supplement: Figure S5 — Effect of size iteration for the estimation of H and HD in the ‘Hispanic’ dataset. Only the mtSNPs overlapping in all the population datasets were used. (0.24 MB TIF) [file pone.0010218.s005.tif]
